# Additional New Minor Cucurbitane Glycosides from *Siraitia grosvenorii*

**DOI:** 10.3390/molecules19033669

**Published:** 2014-03-24

**Authors:** Indra Prakash, Venkata Sai Prakash Chaturvedula

**Affiliations:** 1 The Coca-Cola Company, Organic Chemistry Department, Global Research and Development, One Coca-Cola Plaza, Atlanta, GA 30313, USA; 2 Natural Ingredient Development, Blue California, 30111 Tomas, Rancho Santa Margarita, CA 92688, USA; E-Mail: saipc@bluecal-ingredients.com

**Keywords:** *Siraitia grosvenorii*, curcubitaceae, Luo Han Guo, curcubitane glycosides, structure elucidation, NMR, MS

## Abstract

Continuous phytochemical studies of the crude extract of Luo Han Guo (*Siraitia grosvenorii*) furnished three additional new cucurbitane triterpene glycosides, namely 11-deoxymogroside V, 11-deoxyisomogroside V, and 11-deoxymogroside VI. The structures of all the isolated compounds were characterized on the basis of extensive NMR and mass spectral data as well as hydrolysis studies. The complete ^1^H- and ^13^C-NMR spectral assignments of the three unknown compounds are reported for the first time based on COSY, TOCSY, HSQC, and HMBC spectroscopic data.

## 1. Introduction

The fruit of *Siraitia gros*v*enorii* (*Momordica gros*v*enorii*; Cucurbitaceae), known as Luo Han Guo, has been used for centuries in Traditional Chinese medicine for the treatment of dry cough, sore throat, and minor stomach and intestinal troubles [1,2,3,4]. An intriguing characteristic of Luo Han Guo that is well known now throughout the world is its intense sweet taste and it has been used as a non-caloric natural sweetener in some countries [5]. The extract from this fruit has been reported to be about 150 times sweeter than sucrose. The non-caloric sweet taste of *S. grosvenorii* fruit results primarily from the content of a group of cucurbitane-type triterpene glycosides also known as mogrosides that are present at about 1% in the flesh of the fruit. Early chemical investigation of Luo Han Guo resulted in the isolation of various triterpenoid glycosides and polyphenols [1,2,3,4,5]. 

**Figure 1:**
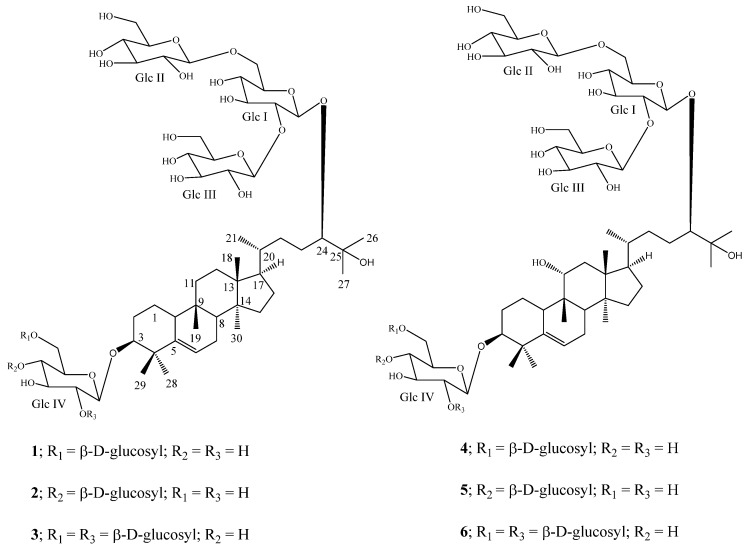
Structures of 11-deoxymogroside V (**1**), 11-deoxyisomogroside V (**2**), 11-deoxymogroside VI (**3**), mogroside V (**4**), isomogroside V (**5**), and mogroside VI (**6**).

In continuation of our study on the isolation of natural sweeteners from *S. grosvenorii* [6,7,8,9], we have investigated a commercial aqueous alcoholic extract of Luo Han Guo, which resulted in the isolation of three new curcubitane glycosides, 11-deoxymogroside V (**1**), 11-deoxyisomogroside V (**2**), and 11-deoxymogroside VI (**3**) (Figure 1). This paper describes the isolation, purification, and structure elucidation of these three new mogrosides **1**–**3** based on the extensive NMR and mass spectroscopic as well as hydrolysis studies. The complete assignments of the ^1^H- and ^13^C-NMR values of the isolated compounds were made on the basis of COSY, TOCSY, HSQC, and HMBC spectral data as well as comparison with the spectral data of known compounds **4**–**6**. 

## 2. Results and Discussion

**Figure 2:**
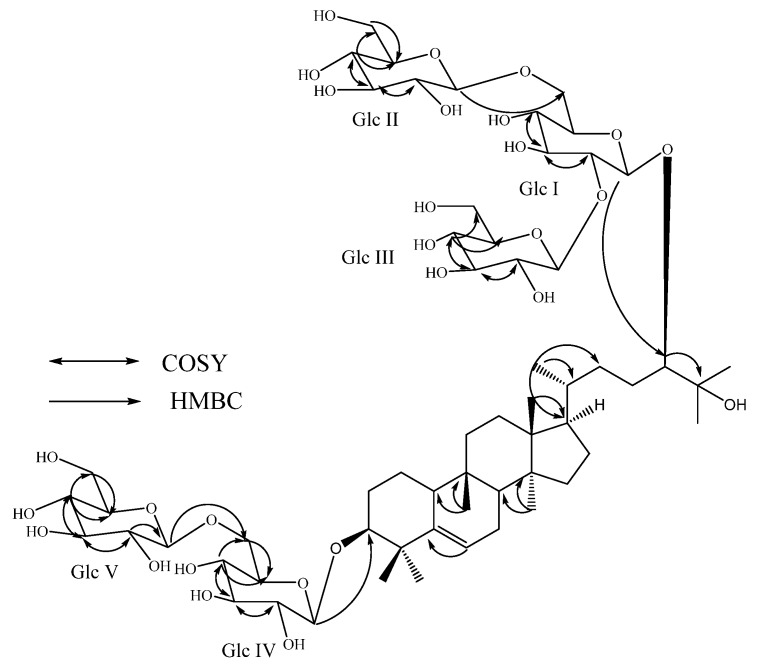
Key COSY and HMBC correlations of 11-deoxymogroside V (**1**).

Compound **1** was isolated as a white powder and its molecular formula was deduced as C_60_H_102_O_28 _from the positive ESI Time of Flight (TOF) mass spectrum (MS) studies which showed an [M+H]^+^ ion at *m/z* 1,271.6649. The ^1^H-NMR spectrum of 1 showed the presence of seven methyl singlets at δ 0.84, 0.85, 0.91, 1.09, 1.39, 1.51, and 1.52; a secondary methyl signal as a doublet at 1.11 (*J* = 6.0 Hz); nine sp^3^ methylene and five sp^3^ methine groups between δ 1.09–2.54. The ^1^H-NMR spectrum of **1** also showed the presence of two oxymethine protons at δ 3.73 and 3.80; an olefinic proton corresponding to a trisubstituted double bond at δ 5.49. The ^13^C-NMR spectrum of 1 showed a tertiary hydroxy group resonating at δ_C_ 72.9. The above spectral data is characteristic to the aglycone moiety of the triterpenoid mogrol, isolated earlier from *S. grosvenorii* [5,6]. The ^1^H-NMR spectrum of **1** also showed the presence of five anomeric protons at δ 4.84, 4.85, 4.93, 5.18, and 5.56, indicating the presence of five hexose moieties. This was confirmed by the MS/MS spectrum of **1**, selecting the [M-H]^−^ ion at *m/z* 1,269 for fragmentation, indicated the loss of five sugar moieties at *m/z* 1,107.5968, 945.5433, 783.4903, 621.4369, and 459.3816 having a difference of 162 atomic mass units, indicative of the presence of these five hexose moieties. Acid hydrolysis of **1** with 5% H_2_SO_4_ afforded glucose, which was identified by direct comparison with an authentic sample by TLC [10,11,12], suggested the presence of five glucosyl moieties in its structure. The configuration of the sugars present in **1** was identified by preparing the corresponding thiocarbamoyl-thiazolidine carboxylate derivatives and comparison of their retention times with the standard sugars as described in the literature [13]. The placement of the five sugar units in **1** was confirmed as 1→6 linked β-D-glucobiosyl substituent at C-3 and 2,6-branched β-D-glucotriosyl moiety at C-24 were identified on the basis of the key COSY, and HMBC correlations as shown in Figure 2. 

The large coupling constants observed for the five D-glucose anomeric protons suggested the β-orientation, as reported for mogrol glycosides. A series of 1D TOCSY experiments selecting each anomeric proton resonating at δ 4.84, 4.85, 4.93, 5.18, and 5.56 together with their HMBC correlations helped in assigning the ^1^H-NMR spectral data for the sugar units in **1**. The ^1^H- and ^13^C-NMR values for all the protons and carbons were assigned on the basis of COSY, TOCSY, HSQC and HMBC correlations and are given in Table 1. A close comparison of the NMR and MS spectral data of **1** with mogroside V (**4**) [6] suggested that the structure of **1** is identical to 4 except for the absence of the 11-hydroxyl group. The stereochemistry of various chiral centers in **1** was considered similar to 4 based on identical ^1^H- and ^13^C-NMR values and multiplicities at the corresponding positions [6,14,15,16]. Thus, the structure of **1** was deduced as 11-deoxymogroside V.

**Table 1:** ^1^H- and ^13^C-NMR chemical shift values (δ, ppm) for the compounds **1**–**3** in pyridine-*d*_5_/D_2_O (10:1) ^a–c^.

Position	1		2		3	
	^1^H	^13^C	^1^H	^13^C	^1^H	^13^C
1	1.62 m 1.85 m	22.6	1.51 m 1.80 m	22.6	1.61 m 1.92 m	22.5
2	1.97 m 2.54 br d (12.0)	28.9	1.91 m 2.38 br d (12.0)	28.9	1.97 m 2.43 br d (11.0)	28.8
3	3.73 br s (*W*_1/2_ = 7.5)	87.5	3.65 br s (*W*_1/2_ = 6.8)	87.7	3.72 br s (*W*_1/2_ = 7.1)	86.6
4	---	41.9	---	42.4	---	41.5
5	---	143.4	---	143.3	---	142.9
6	5.49 d (4.2)	118.8	5.50 m	118.9	5.70 br s	119.2
7	1.64 m 2.24 m	24.4	1.65 m 2.24 m	24.5	1.67 m 2.43 m	24.7
8	1.62 m	43.6	1.62 m	43.8	1.59 m	43.8
9	---	35.1	---	36.6	---	34.4
10	2.27 m	38.3	2.28 m	38.4	2.28 m	38.2
11	1.36 m 1.59 m	32.4	1.37 m 1.58 m	32.6	1.35 m 1.62 m	32.5
12	1.45 m 1.62 m	30.6	1.46 m 1.65 m	30.8	1.44 m 1.63 m	30.9
13	---	46.7	---	46.5	---	46.1
14	---	49.4	---	49.7	---	49.3
15	1.09 m 1.19 m	34.9	1.09 m 1.19 m	35.0	1.08 m 1.20 m	35.1
16	1.49 m 2.16 m	28.2	1.48 m 2.15 m	28.4	1.50 m 2.16 m	28.5
17	1.69 m	50.8	1.68 m	50.9	1.69 m	50.8
18	0.85 s	15.5	0.85 s	15.7	0.84 s	15.8
19	0.91 s	28.1	0.88 s	28.2	0.99 s	28.3
20	1.57 m	36.2	1.56 m	36.3	1.57 m	36.2
21	1.11 d (6.0)	19.1	1.10 d (6.3)	19.2	1.10 d (6.1)	19.3
22	1.76 m 2.00 m	33.3	1.76 m 1.99 m	33.4	1.75 m 2.00 m	33.3
23	1.66 m 1.97 m	29.0	1.65 m 1.93 m	29.1	1.67 m 1.94 m	29.0
24	3.80 d (9.0)	92.4	3.80 d (8.9)	92.6	3.80 d (9.4)	92.6
25	---	72.9	---	73.2	---	72.8
26	1.39 s	26.7	1.39 s	26.9	1.39 s	26.9
27	1.52 s	24.4	1.51 s	24.5	1.52 s	24.6
28	1.09 s	28.2	1.11 s	28.3	1.06 s	28.2
29	1.51 s	25.6	1.52 s	25.8	1.50 s	25.9
30	0.84 s	18.0	0.83 s	18.2	0.85 s	18.2
Glc-1						
1	4.93 d (6.8)	103.7	4.92 d (6.8)	103.8	4.93 d (7.1)	103.8
2	4.29 m	81.1	4.27 m	81.2	4.29 m	81.1
3	4.30 m	78.3	4.29 m	78.4	4.29 m	78.2
4	3.98 m	71.0	3.97 m	71.1	3.98 m	71.2
5	4.10 m	76.1	4.09 m	76.2	4.09 m	76.3
6	3.98 m 4.89 d (9.7)	69.9	3.97 m 4.88 d (9.4)	70.0	3.98 m 4.89 d (9.5)	70.0
Glc-2						
1	4.850 d (7.3)	104.5	4.85 d (7.7)	104.6	4.850 d (7.7)	104.5
2	4.06 m	74.9	4.06 m	75.0	4.06 m	75.0
3	4.27 m	77.6	4.28 m	77.6	4.29 m	78.2
4	4.18 m	71.3	4.18 m	71.4	4.18 m	71.5
5	3.95 m	78.0	3.94 m	78.2	3.93 m	78.2
6	4.30 m 4.50 d (11.0)	62.2	4.30 m 4.50 m	62.2	4.30 m 4.50 dd (1.9, 12.0)	62.4
Glc-3						
1	5.56 d (7.9)	104.6	5.18 d (7.7)	104.8	5.56 m	104.7
2	4.11 m	75.6	4.08 m	74.7	4.11 m	75.8
3	4.23 m	77.9	4.27 m	77.8	4.23 m	78.0
4	4.07 m	71.9	4.16 m	71.4	4.07 m	72.2
5	4.00 m	78.1	4.02 m	78.3	4.01 m	78.2
6	4.32 m 4.60 d (12.0)	63.1	4.23 m 4.53 m	62.2	4.32 m 4.60 dd (2.3, 12.0)	63.3
Glc-4						
1	4.845 d (7.3)	106.6	4.84 d (7.7)	106.7	4.86 d (7.7)	104.5
2	3.95 m	75.1	3.98 m	74.8	4.28 m	81.3
3	4.23 m	77.9	4.27 m	76.5	4.29 m	78.2
4	4.13 m	77.0	4.27 m	81.4	3.98 m	71.2
5	4.06 m	74.9	3.92 m	76.2	4.07 m	76.8
6	4.35 m 4.83 m	69.9	4.30 m 4.50 m	62.1	4.32 m 4.78 m	69.8
Glc-5						
1	5.18 d (7.9)	104.9	5.57 d (8.1)	104.8	5.34 d (7.7)	104.7
2	4.06 m	74.9	4.10 m	75.8	4.09 m	76.8
3	4.31 m	78.0	4.23 m	77.8	4.24 m	77.7
4	4.19 m	71.3	4.07 m	72.2	4.30 m	71.3
5	4.00 m	78.1	4.00 m	78.3	3.92 m	78.2
6	4.35 m 4.54 d (12.0)	62.5	4.31 m 4.59 m	63.2	4.46 m 4.55 m	62.5
Glc-6						
1					5.16 d (7.8)	105.0
2					4.07 m	74.9
3					4.29 m	78.2
4					4.19 m	71.5
5					4.01 m	78.3
6					4.35 m4.57 m	62.6

^a^ Assignments were made on the basis of COSY, TOCSY, HSQC and HMBC spectral data; ^b^ Coupling constants are in Hz; ^c^ Chemical shift values are in δ (ppm); Glc: β-D-glucosyl.

**Figure 3:**
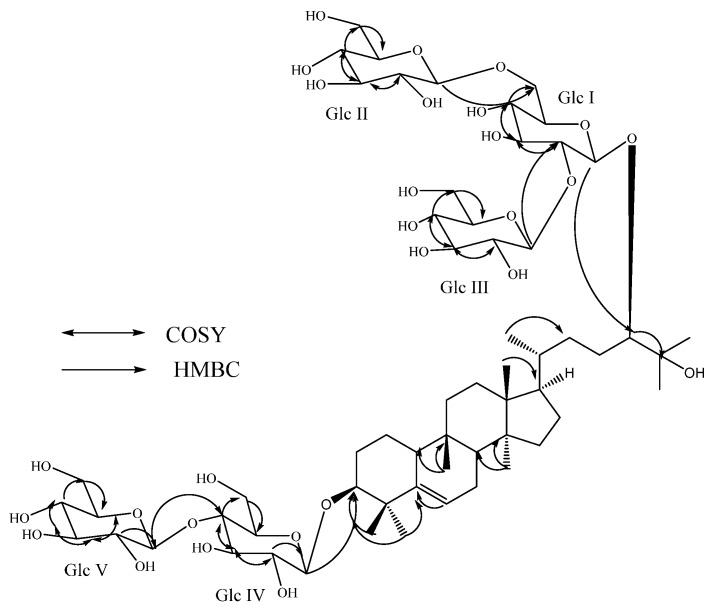
Key COSY and HMBC correlations of 11-deoxyisomogroside V (**2**).

Compound **2**, was also isolated as a white amorphous powder and its molecular formula was inferred as C_60_H_102_O_28_ from the ESI TOF MS data, suggesting its molecular weight as 1,270; identical to **1**. The ^1^H-NMR spectrum of **2** showed the presence of seven methyl singlets at δ 0.83, 0.85, 0.88, 1.11, 1.39, 1.51, and 1.52; a secondary methyl signal as doublet at δ 1.10 (*J* = 6.3 Hz); nine methylene and four methine groups between δ 1.09–2.38; two oxymethine protons at δ 3.65 and 3.80; an olefinic proton corresponding to a trisubstituted double bond at δ 5.50; five anomeric protons at 4.84, 4.85, 4.92, 5.18, and 5.57; a tertiary hydroxy group resonating at δ_C_ 73.2. Acid hydrolysis of 2 with 5% H_2_SO_4_ also afforded glucose, which was identified by direct comparison with an authentic sample by co-TLC [10,11,12] and its configuration was identified as D, similar to **1**. The large coupling constants observed for the five D-glucose anomeric protons suggested the β-orientation as in **1** and other mogrosides [5,6]. From the above spectral and chemical studies, compound **2** was found to be similar to compound **1** in its structure by having identical chemical shifts for the aglycone moiety in its ^1^H-NMR spectral data and five glucosyl moieties. Based on the key COSY and HMBC correlations of **2** as shown in Figure 3, it was found that three glucosyl units are present at C-24 as 2,6-branched β-D-glucotriosyl moiety similar to **1**, whereas the additional two glucosyl units at C-3 were attached as 1→4 linked β-D-glucobiosyl substituent suggesting a structure identical to that of isomogroside V (**5**) [17], except for the absence of the 11-hydroxyl group. This was supported by the MS data of **2** which showed 16 atomic mass units less for **5**. The ^1^H- and ^13^C-NMR values for all the protons and carbons were assigned on the basis of COSY, TOCSY, HSQC and HMBC correlations and are given in Table 1. Considering the stereochemistry at all chiral centers similar to isomogroside V (**5**) [17] by the identical ^1^H- and ^13^C-NMR values as well as in comparison with reported values for various cucurbitane glycosides [6,14,15,16], the structure of **2** was assigned as 11-deoxyisomogroside V.

**Figure 4:**
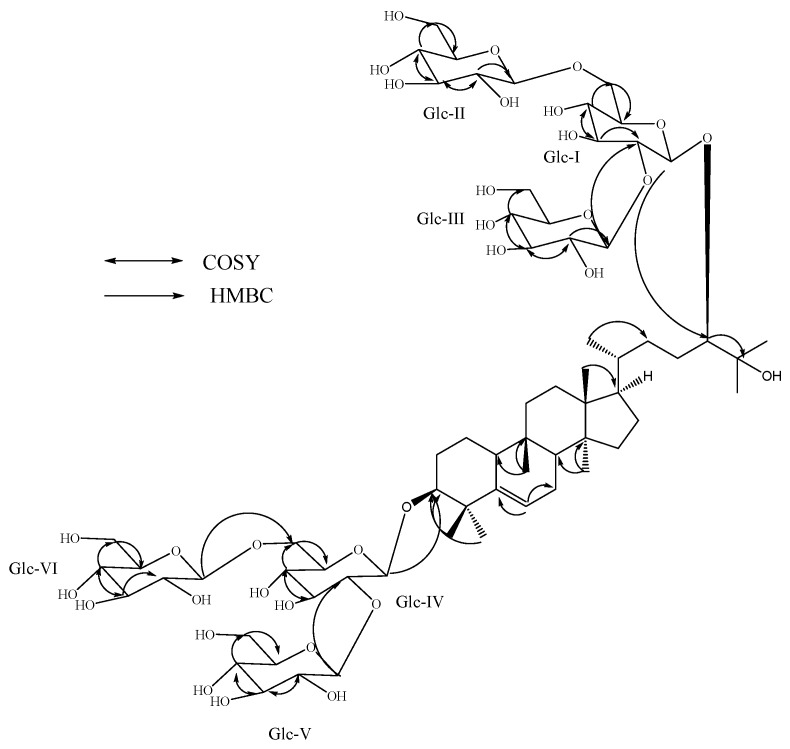
Key COSY and HMBC correlations of 11-deoxymogroside VI (**3**).

Compound **3**, was also obtained as a white amorphous powder. The positive ESI TOF mass spectrum of **3** showed an [M+H]^+^ ion at *m/z* 1,433.7169 which was in good agreement with the molecular formula C_66_H_112_O_33_. The ^1^H- and ^13^C-NMR spectrum of **3** showed seven methyl singlets; a secondary methyl signal as doublet; nine methylene and five methine groups; two oxymethine protons; an olefinic proton corresponding to a trisubstituted double bond; a tertiary hydroxy group similar to the aglycone moiety peaks observed in **1** and **2**. The MS/MS spectrum of **3**, selecting the [M–H]^−^ ion at *m/z* 1431 for fragmentation, indicated the loss of six sugar moieties at *m/z* 1269.6481, 1107.5962, 945.5418, 783.4893, 621.4377, and 459.3869, and further the difference of 162 mass units suggests the presence of six hexose moieties. This was also supported by the presence of anomeric protons resonating at δ 4.85, 4.86, 4.93, 5.16, 5.34, and 5.56 in its ^1^H-NMR spectral data. Acid hydrolysis of **3** that was performed as in **1** and **2** confirmed the sugars as D-glucose and the large coupling constants observed for the six D-glucose anomeric protons suggested their β-orientation. The above spectral and hydrolysis studies indicated that the structure of **3** has six β-D-glucotriosyl units on the aglycone moiety presented in compounds **1** and **2**. The presence of a 2,6-branched β-D-glucotriosyl moieties at the C-24 and C-3 positions in **3** was supported on the basis of the key COSY and HMBC correlations shown in Figure 4 and in comparison of its NMR values with **1** and mogroside VI (**6**) [6]. The ^1^H- and ^13^C-NMR values for all the protons and carbons were assigned on the basis of COSY, TOCSY, HSQC and HMBC correlations and are given in Table 1, suggesting the stereochemistry at all chiral centers identical to compounds **1** and **2** [6,14,15,16]. Thus, the structure of **3** was confirmed as 11-deoxy- mogroside VI. 

## 3. Experimental

### 3.1. General

NMR spectra were acquired on a Bruker Avance DRX 500 MHz instrument with a 5 mm inverse detection probe using standard pulse sequences. The spectrum was referenced to the residual solvent signal (δ_H_ 8.71, δ_C_ 149.9 for pyridine-*d*_5_), chemical shifts are given in δ (ppm), and coupling constants are reported in Hz, MS and MS/MS data were generated with a Waters Premier Quadrupole Time-of-Flight (Q-Tof) mass spectrometer equipped with an electrospray ionization source operated in the positive-ion mode and ThermoFisher Discovery OrbiTrap in the positive Positive Mode Electrospray. Samples were diluted with water: acetonitrile (1:1) containing 0.1% formic acid and introduced via infusion using the onboard syringe pump. The details of various columns and other parameters used for HPLC purification for methods 1–6 are given below:

Method 1; Column: Phenomenex Gemini NX C_18_, 250 × 21.2 mm, 5 μm (p/n 00G-4097-P0) with a Phenomenex guard column; UV Detection: 210 nm; Mobile Phase A: water; Mobile Phase B: Acetonitrile; Flow Rate: 20 mL/min.

Methods 2 and 5: Column: Phenomenex Synergi Hydro RP, 250 × 10 mm, 4 μm (p/n 00G-4375-N0) with a Phenomenex guard column; UV Detection: 210 nm; Mobile Phase A: water; Mobile Phase B: Acetonitrile; Flow Rate: 5 mL/min.

Methods 3 and 6: Column: ZIC-HILIC, 250 × 10 mm, 5 μm (p/n HX 129616) with a Phenomenex guard column; UV Detection: 210 nm; Mobile Phase A: water; Mobile Phase B: Acetonitrile; Flow Rate: 5 mL/min.

Method 4: Column: Waters Atlantis Prep T3, 250 × 10 mm, 5 μm (p/n 186003694) with a Phenomenex guard column; UV Detection: 210 nm; Mobile Phase A: water; Mobile Phase B: Acetonitrile; Flow Rate: 5 mL/min.

### 3.2. Plant Material

The Luo Han Guo commercial extract was purchased from Chengdu Biopurify Phytochemicals, Chengdu, Sichuan, China. A voucher specimen is deposited at The Coca Cola Company, No. VSPC-3166-99.

### 3.3. Isolation

An aliquot of commercial crude extract of mogrosides (15 g, 90% mogrosides) was taken in a round-bottomed flask and MeOH (60 mL) was added. The mixture was stirred for 15 min and heated to reflux. After refluxing the mixture for 1 h, it was cooled to RT and stirring was continued for an additional 24 h after the addition of acetone (300 mL). The solid formed was filtered out and concentrated the filtrate concentrated to obtain a residue (2.3 g). A series of HPLC purifications were performed on this residue (Table 2) to obtain the three new mogrosides. A first round of HPLC purification was performed using method 1 and fractions eluted between 19 and 21 min were collected, which on evaporation under vacuum furnished Fraction 1. A second round of purification of Fraction 1 was performed using method 2 and fractions collected between 6.5 and 8.5 min, as well as 19 and 23 min were collected, which on concentration yielded Fractions 2 and 3, respectively. Purification of Fraction 2 using HPLC method 3, collection of the elute at 16.4 min and concentration, followed by a final round of purification using HPLC method 4 over multiple runs furnished **3** (*t_R_* 16.4 min, 4.2 mg). Similarly, purification of Fraction **3** using HPLC method 5, collection of the elute between 15 and 17 min, and a final round of purification using HPLC 6 method over multiple runs furnished **1** (4.2 mg), and **2** (3.6 mg).

**Table 2:** RP-HPLC methods for the isolation and purification of cucurbitane glycosides **1**–**3**.

HPLC Method	Time (min)	% of Mobile Phase A	% of Mobile Phase B
Method 1	0.0	90	10
	15.0	75	25
	20.0	54	46
	21.0	10	90
	26.0	10	90
	26.5	90	10
	31.0	90	10
Method 2	0.0	75	25
	15.0	75	25
	16.0	70	30
	30.0	70	30
	31.0	10	90
	36.0	10	90
	37.0	75	25
	45.0	75	25
Method 3	0.0	18	82
	15.0	18	82
	20.0	50	50
	21.0	95	5
	25.0	95	5
	26.0	18	82
	31.0	18	82
Method 4	0.0	75	25
	15.0	75	25
	30.0	65	35
	31.0	30	70
	35.0	30	70
	36.0	75	25
	40.0	75	25
Method 5	0.0	75	25
	8.5	75	25
	10.0	71	29
	16.5	70	30
	18.5	66	34
	24.5	66	34
	26.5	48	52
	29.0	48	52
Method 5	31.0	30	70
	37.0	30	70
	37.1	75	25
	45.0	75	25
Method 6	0.0	15	85
	20.0	15	85
	30.0	95	5
	31.0	95	5
	36.0	15	85
	37.0	15	85

*11-deoxymogroside V* (**1**). White powder, ^1^H-NMR [500 MHz, pyridine-d5/D2O (10:1), δ ppm] and ^13^C-NMR [125 MHz, pyridine-d5/D2O (10:1), δ ppm] data see Table 1; HRMS (M+H)^+^ *m/z* 1271.6649 (calcd. for C_60_H_103_O_28_: 1271.6636).

*11-deoxyisomogroside V* (**2**). White powder; ^1^H-NMR [500 MHz, pyridine-d5/D2O (10:1), δ ppm] and ^13^C-NMR [125 MHz, pyridine-d5/D2O (10:1), δ ppm] data see Table 1; HRMS (M+Na)+ m/z 1293.6447 (calcd. for C_60_H_102_O_28_Na: 1293.6455).

*11-deoxymogroside VI* (**3**). White powder; ^1^H-NMR [500 MHz, pyridine-d5/D2O (10:1), δ ppm] and ^13^C-NMR [125 MHz, pyridine-d5/D2O (10:1), δ ppm] data see Table 1; HRMS (M+H)+ m/z 1433.7169 (calcd. for C_66_H_113_O_33_: 1433.7164).

*Acid Hydrolysis of Compounds* **1**–**3**. To a solution of each compound (2 mg) in MeOH (3 mL) was added 5% H_2_SO_4_ (3 mL) and the mixture was refluxed for 8 h. The reaction mixture was then neutralized with saturated sodium carbonate and extracted with ethyl acetate (EtOAc, 2 × 25 mL) to give an aqueous fraction containing sugars and an EtOAc fraction containing the aglycone part. The aqueous phase was concentrated and compared with standard sugars using the TLC systems EtOAc/*n*-butanol/water (2:7:1) and CH_2_Cl_2_/MeOH/water (10:6:1) [10,11,12]. The sugar was identified as glucose for all three compounds **1**–**3**. 

*General Procedure for Acid Hydrolysis and Determination of Sugar Configuration in* **1**–**3**

Each compound **1**–**3** (1 mg) was hydrolyzed with 0.5 M HCl (0.5 mL) for 1.5 h. After cooling, the mixture was passed through an Amberlite IRA400 column and the eluate was lyophilized. The residue was dissolved in pyridine (0.25 mL) and heated with L-cysteine methyl ester HCl (2.5 mg) at 60 °C for 1.5 h. Then, *O*-tolyl isothiocyanate (12.5 µL) was added to the mixture and heated at 60 °C for an additional 1.5 h. The reaction mixture was analyzed by HPLC: column Phenomenex Luna C18, 150 × 4.6 mm (5 μm); 25% acetonitrile-0.2% TFA water, 1 mL/min; UV detection at 250 nm. The sugar was identified as D-glucose (*t*_R_, 12.42, 12.57 and 12.51 min) in all compounds **1**–**3** [authentic samples, D-glucose (*t*_R_, 12.44) and L-glucose (*t*R, 11.24 min)] [13].

## 4. Conclusions

Three new triterpenoid glycosides **1**–**3** were isolated from a commercial extract of *S. grosvenorii*. The structures of the new compounds were identified as 11-deoxymogroside V, 11-deoxyisomogroside V and 11-deoxymogroside VI, respectively, on the basis of extensive NMR and mass spectroscopic data and chemical studies. To the best of our knowledge this is the first report of the isolation of these three triterpene glycosides from *S. grosvenorii* in Nature. The discovery of these compounds is an important addition in expanding our understanding of the diversity of the triterpenoid glycosides present in the *S. grosvenorii*.

## Supplementary Materials

Supplementary materials can be accessed at: http://www.mdpi.com/1420-3049/19/3/3669/s1.
