# Supplementary material for: Additional New Minor Cucurbitane Glycosides from Siraitia grosvenorii
Source: Molecules. 2014 Mar 24;19(3):3669–80. doi: 10.3390/molecules19033669 (PMC6271671; doi:10.3390/molecules19033669)
Supplement: Supplementary file 1 [file molecules-19-03669-s001.pdf]

Supplementary File

Figure S1. 1D and 2D NMR spectra of 11-deoxymogroside V (**1**). (**a**) ^{1}H-NMR spectrum of **1**; (**b**) ^{1}H-^{1}H COSY spectrum of **1**; (**c**) ^{1}H-^{1}H TOCSY spectrum of **1**; (**d**) ^{1}H-^{13}C HSQC spectrum of **1**; (**e**) ^{1}H-^{13}C HMBC spectrum of **1**.

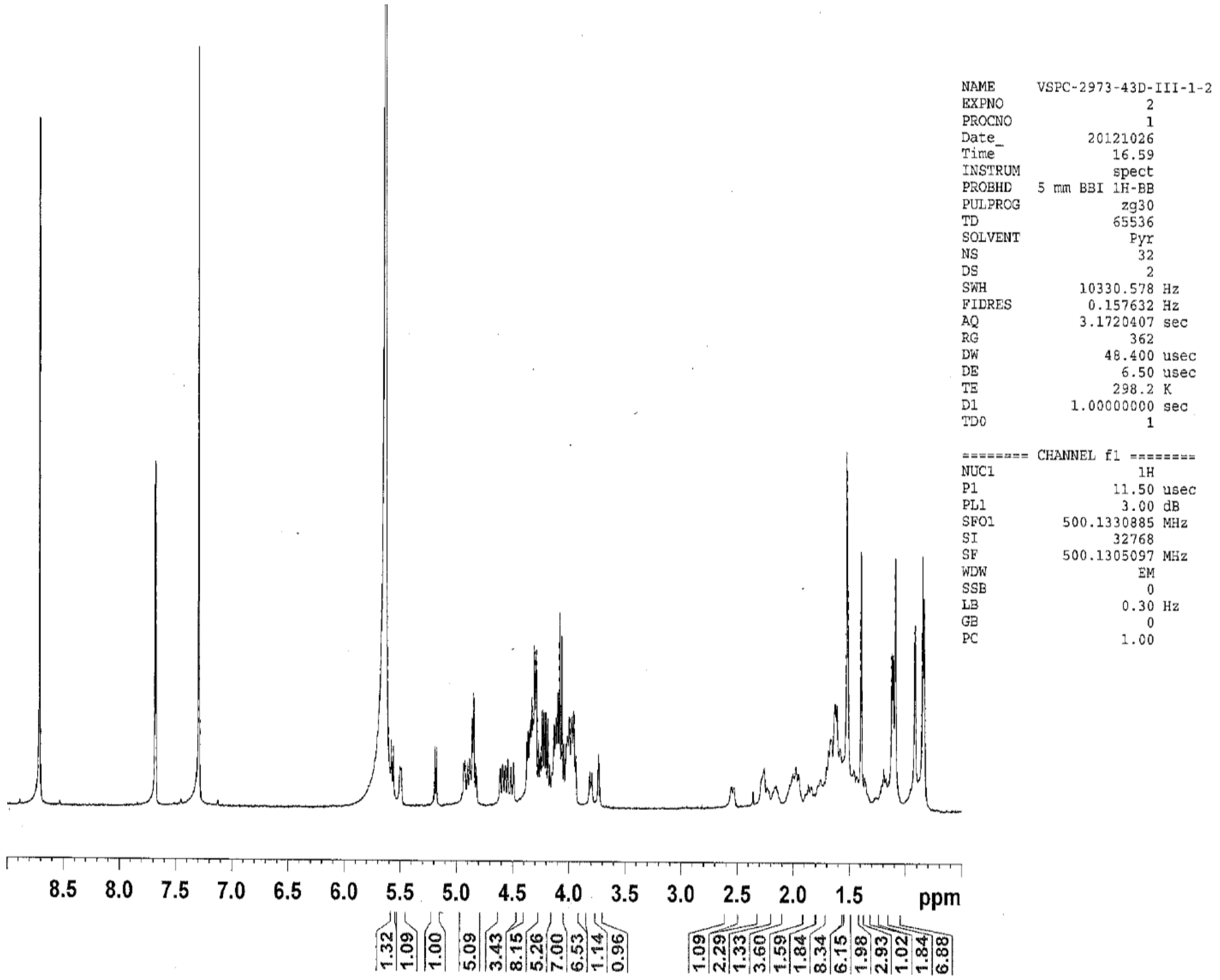

(**a**)

Figure S1. *Cont.*

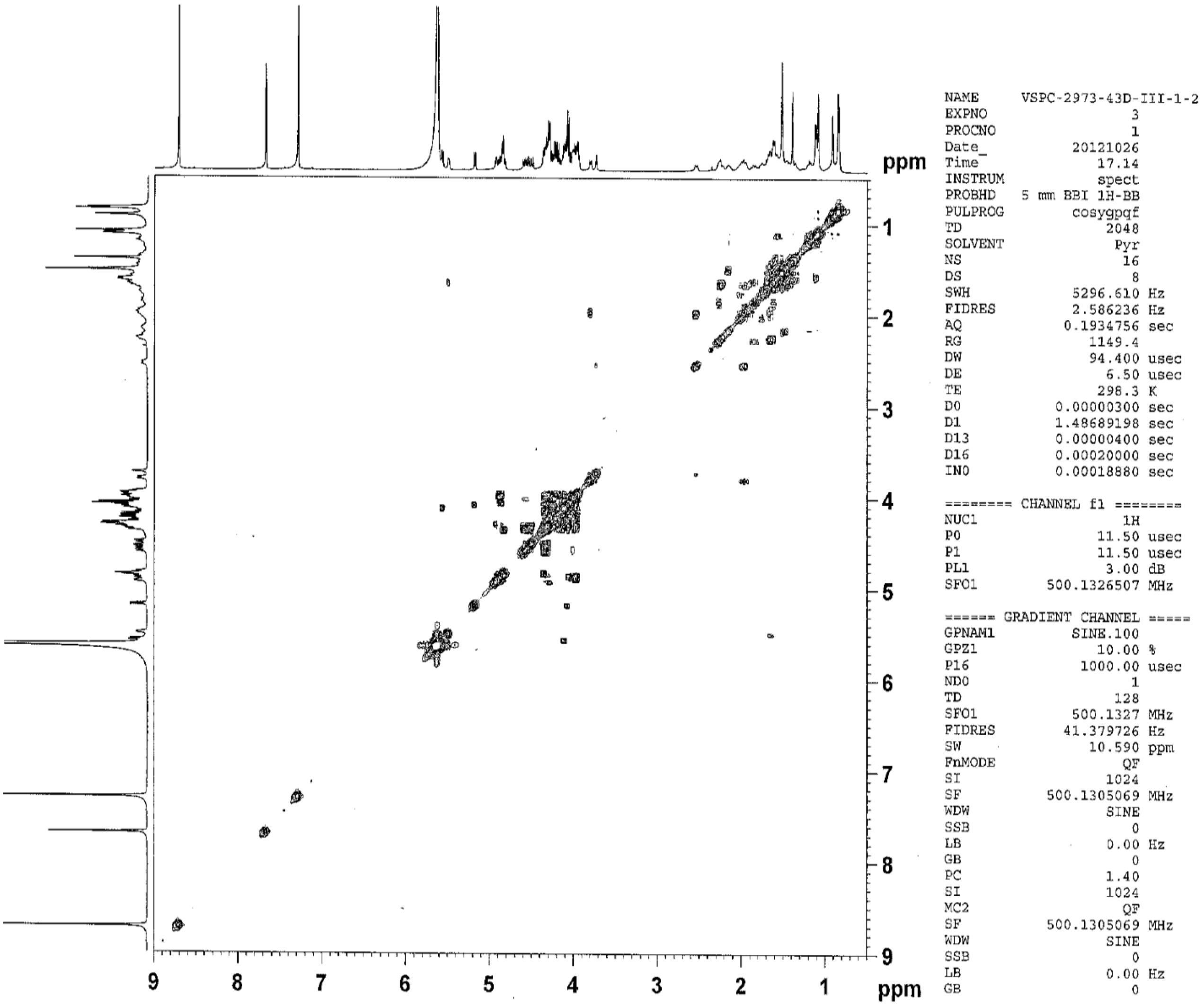

(**b**)

Figure S1. *Cont.*

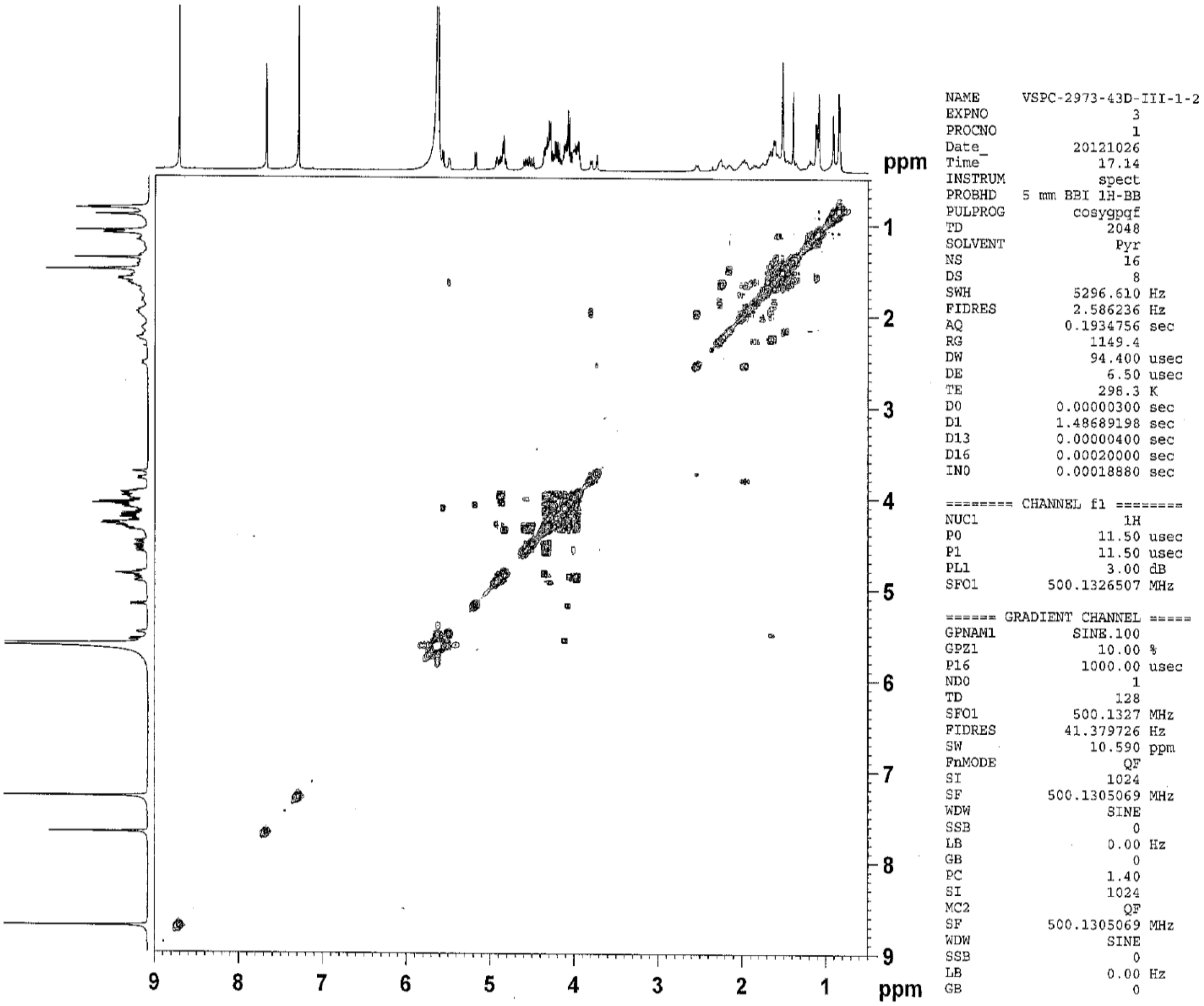

(**c**)

Figure S1. *Cont.*

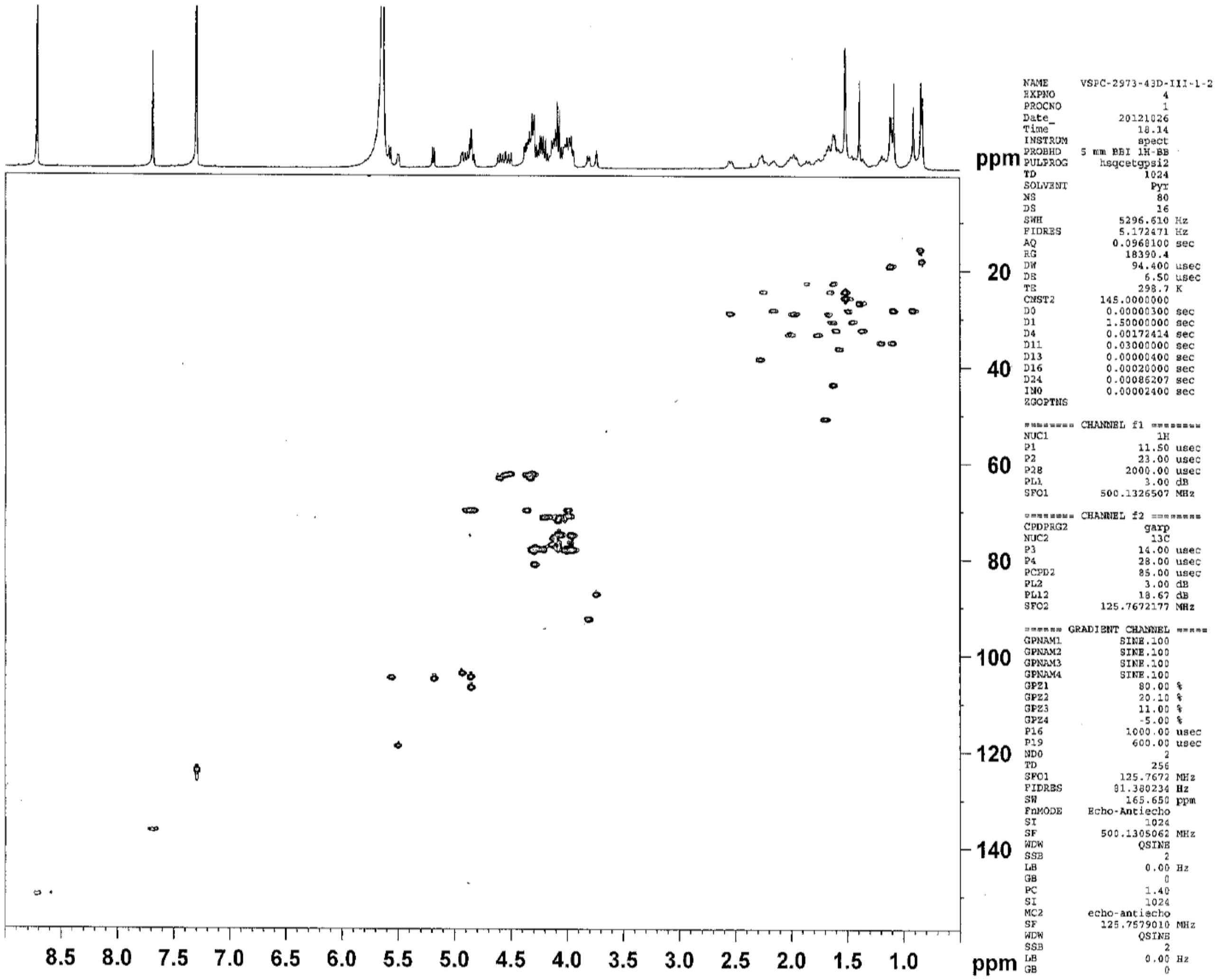

(**d**)

Figure S1. *Cont.*

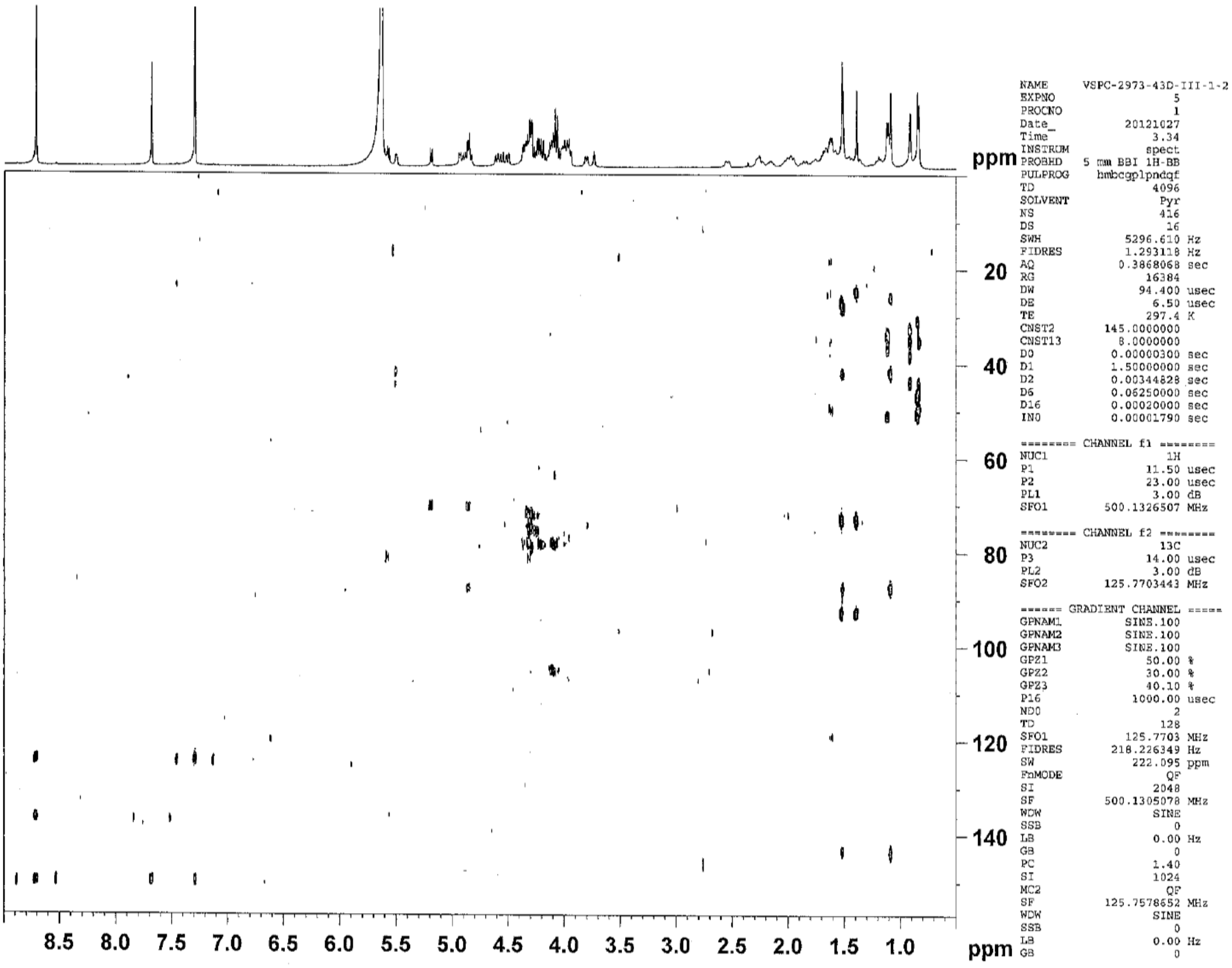

(**e**)

Figure S2. 1D and 2D NMR spectra of 11-deoxyisomogroside V (**2**). (**a**) ^{1}H-NMR spectrum of **2**; (**b**) ^{1}H-^{1}H COSY spectrum of **2**; (**c**) ^{1}H-^{1}H TOCSY spectrum of **2**; (**d**) ^{1}H-^{13}C HSQC spectrum of **2**; (**e**) ^{1}H-^{13}C HMBC spectrum of **2**.

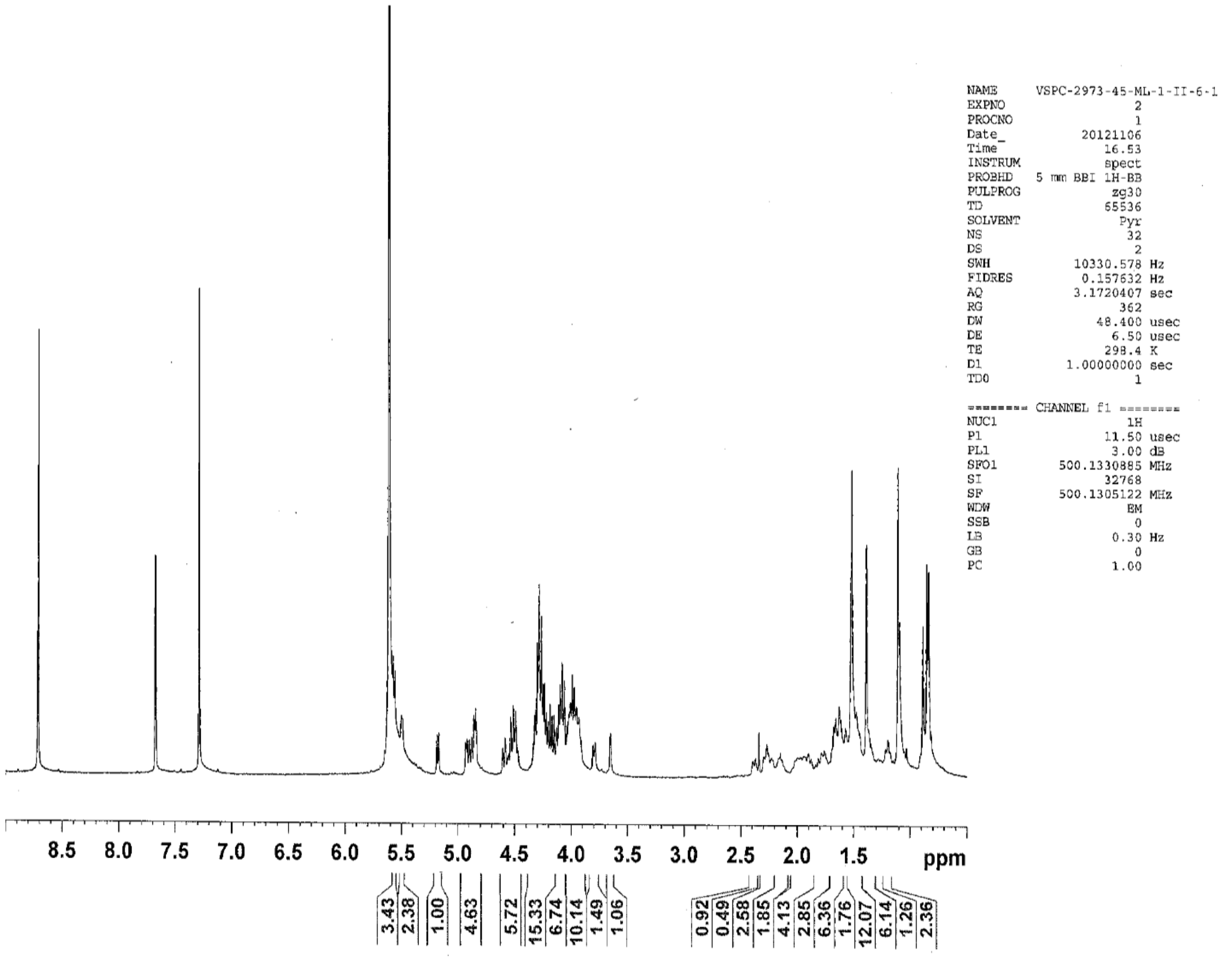

(**a**)

Figure S2. *Cont.*

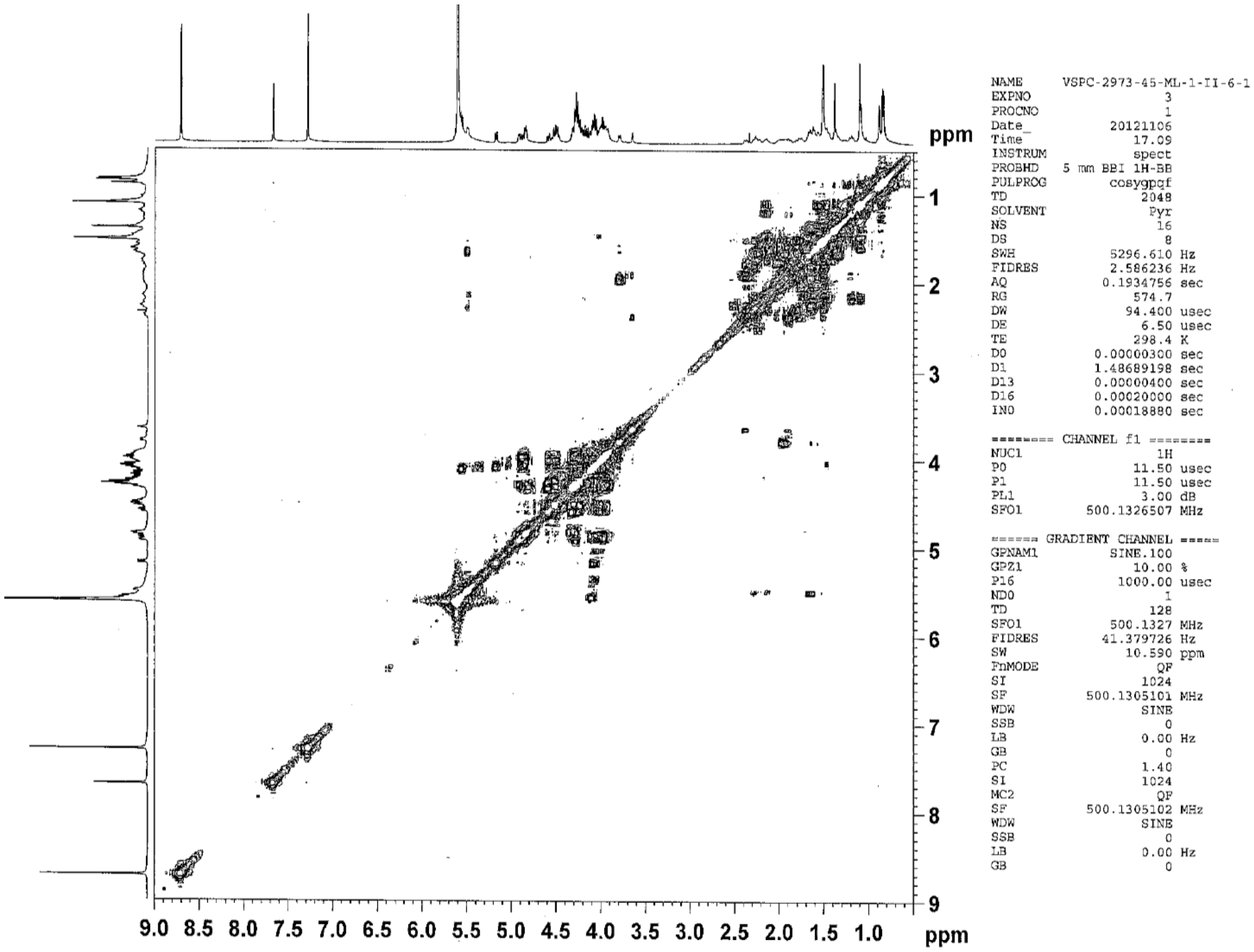

(**b**)

Figure S2. *Cont.*

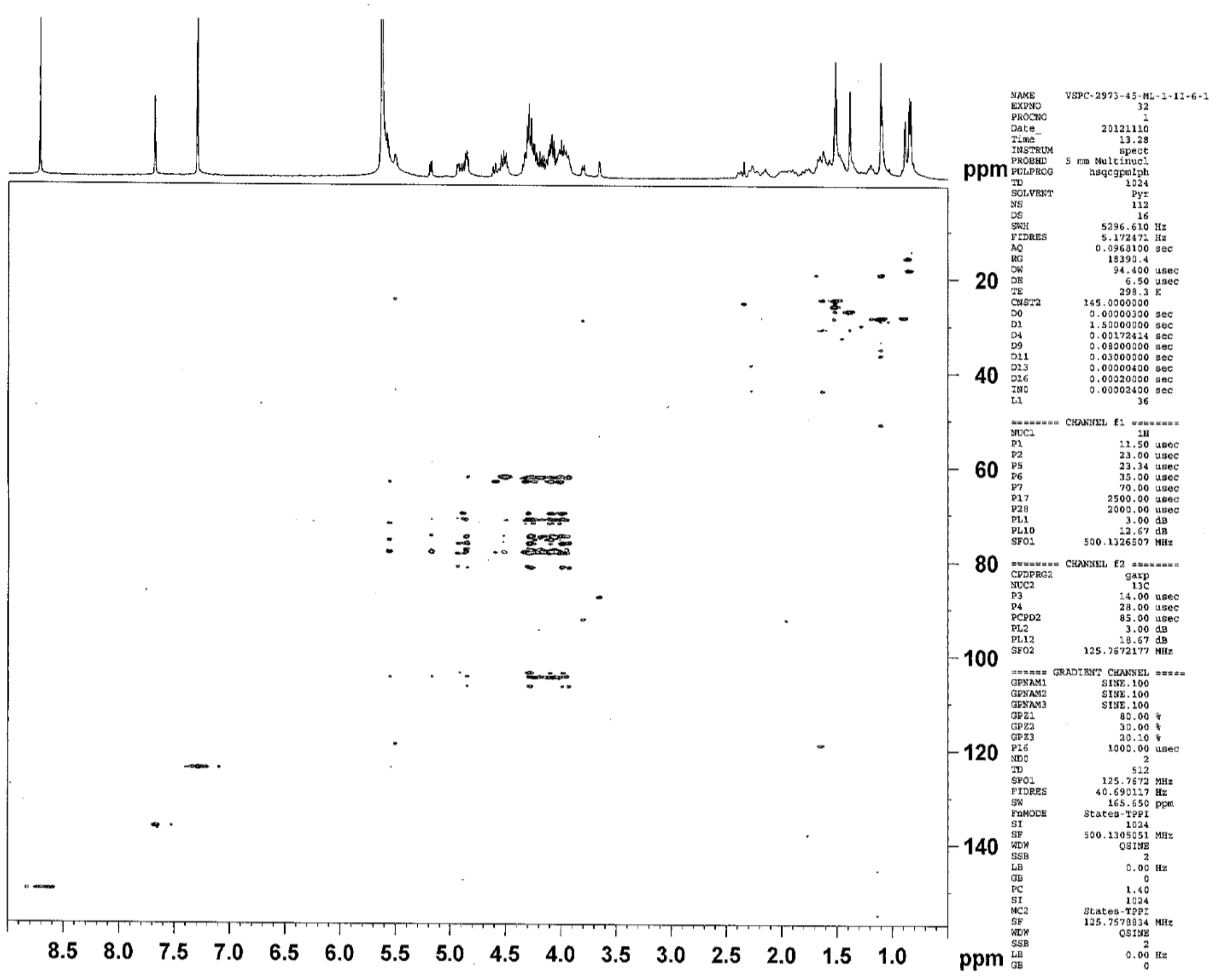

(**c**)

Figure S2. *Cont.*

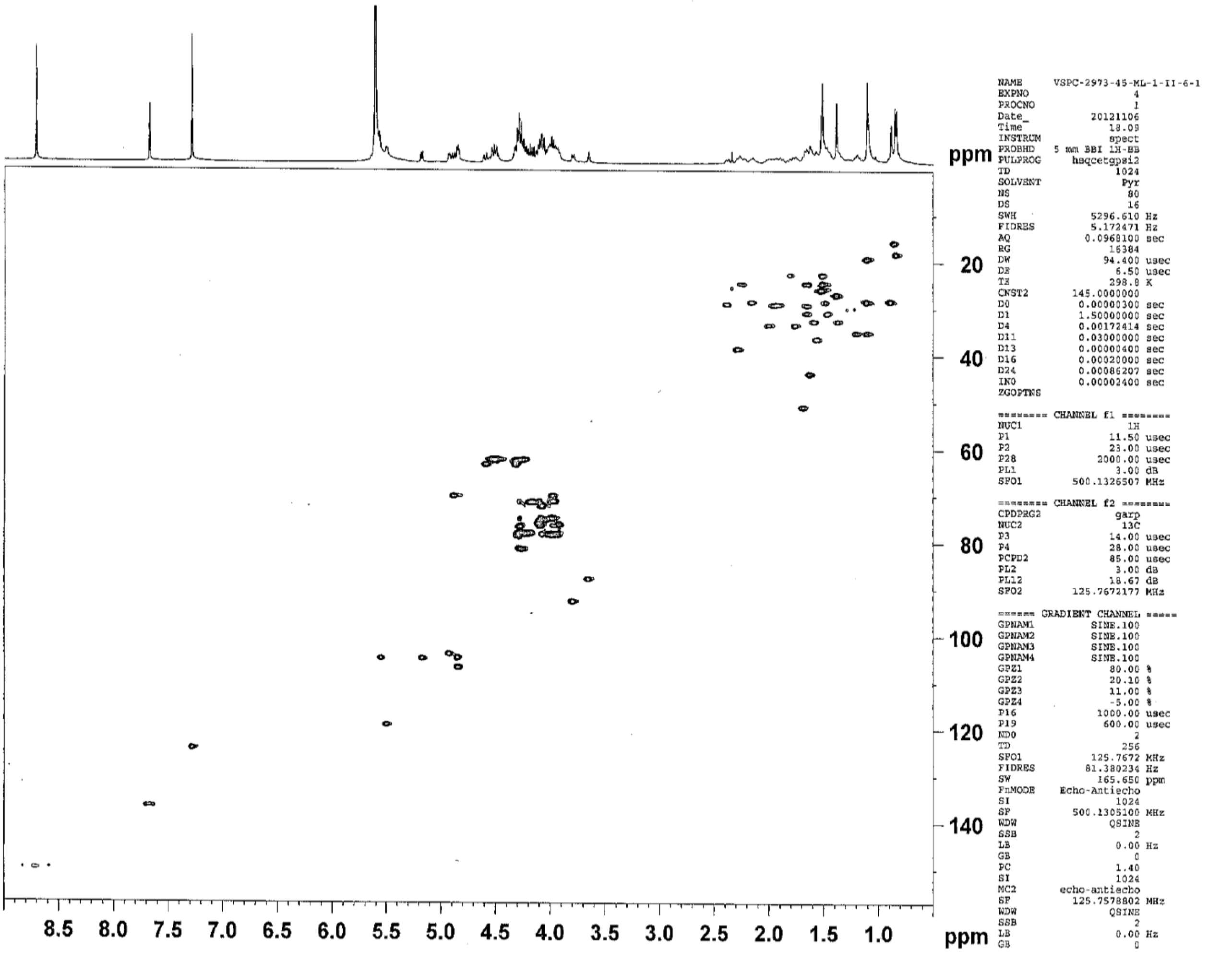

(**d**)

Figure S2. *Cont.*

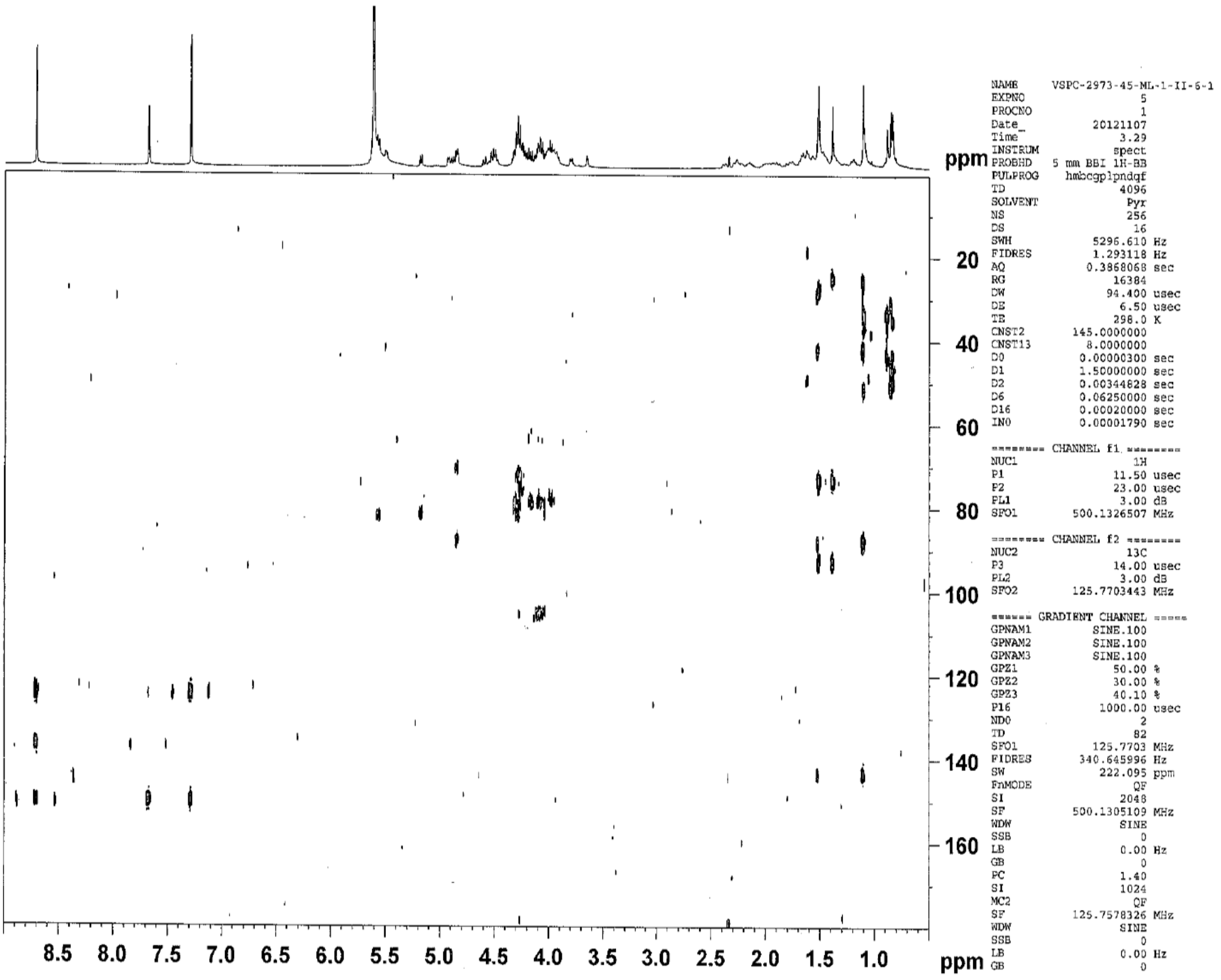

(e)

Figure S3. 1D and 2D NMR spectra of 11-deoxymogroside VI (**3**). (**a**) ^{1}H-NMR spectrum of **3**; ^{1}H-NMR spectrum of **3**; (**b**) ^{1}H-^{1}H COSY spectrum of **3**; (**c**) ^{1}H-^{1}H TOCSY spectrum of **3**; (**d**) ^{1}H-^{13}C HSQC spectrum of **3**. (**e**) ^{1}H-^{13}C HMBC spectrum of **3**.

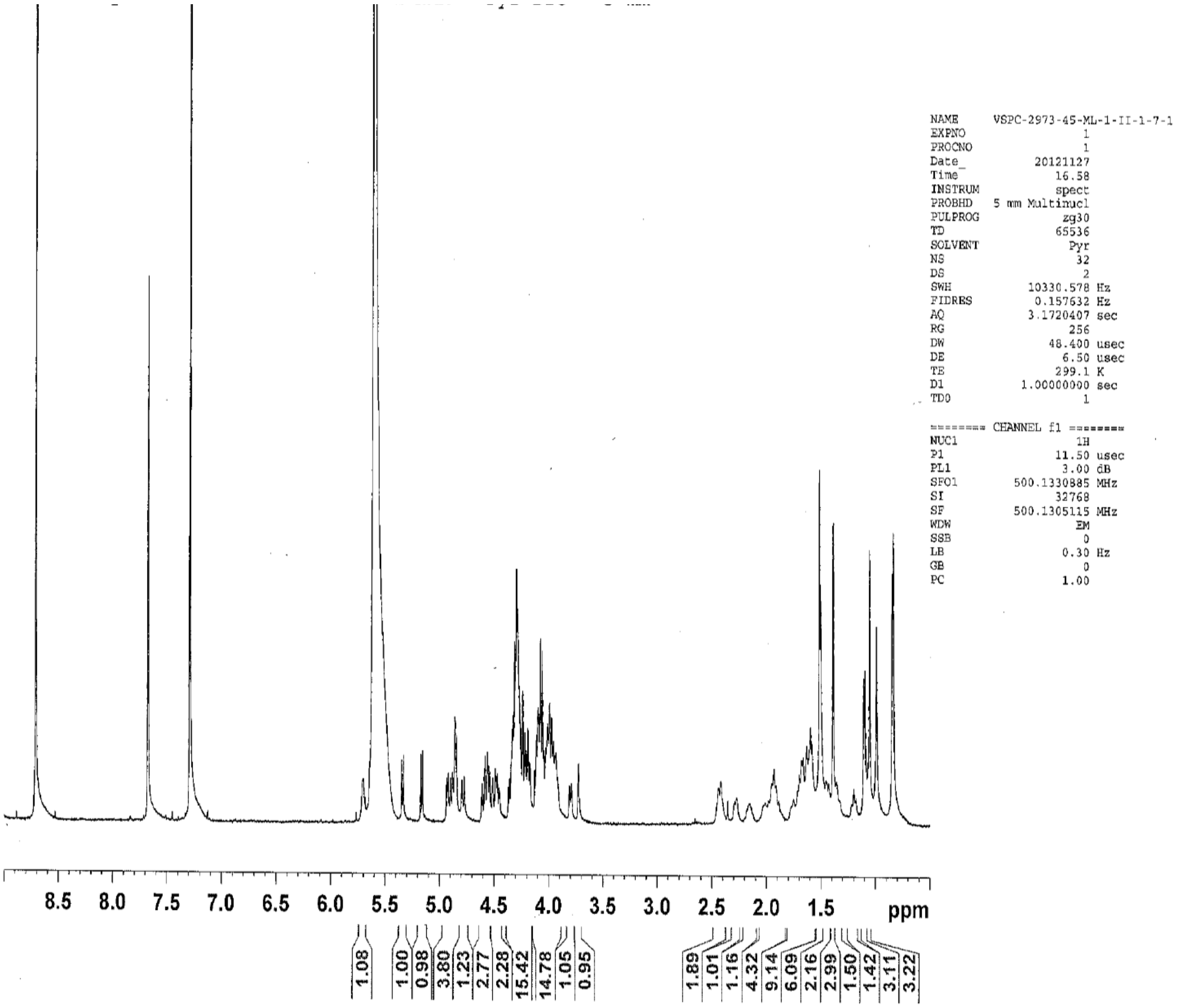

(**a**)

Figure S3. *Cont.*

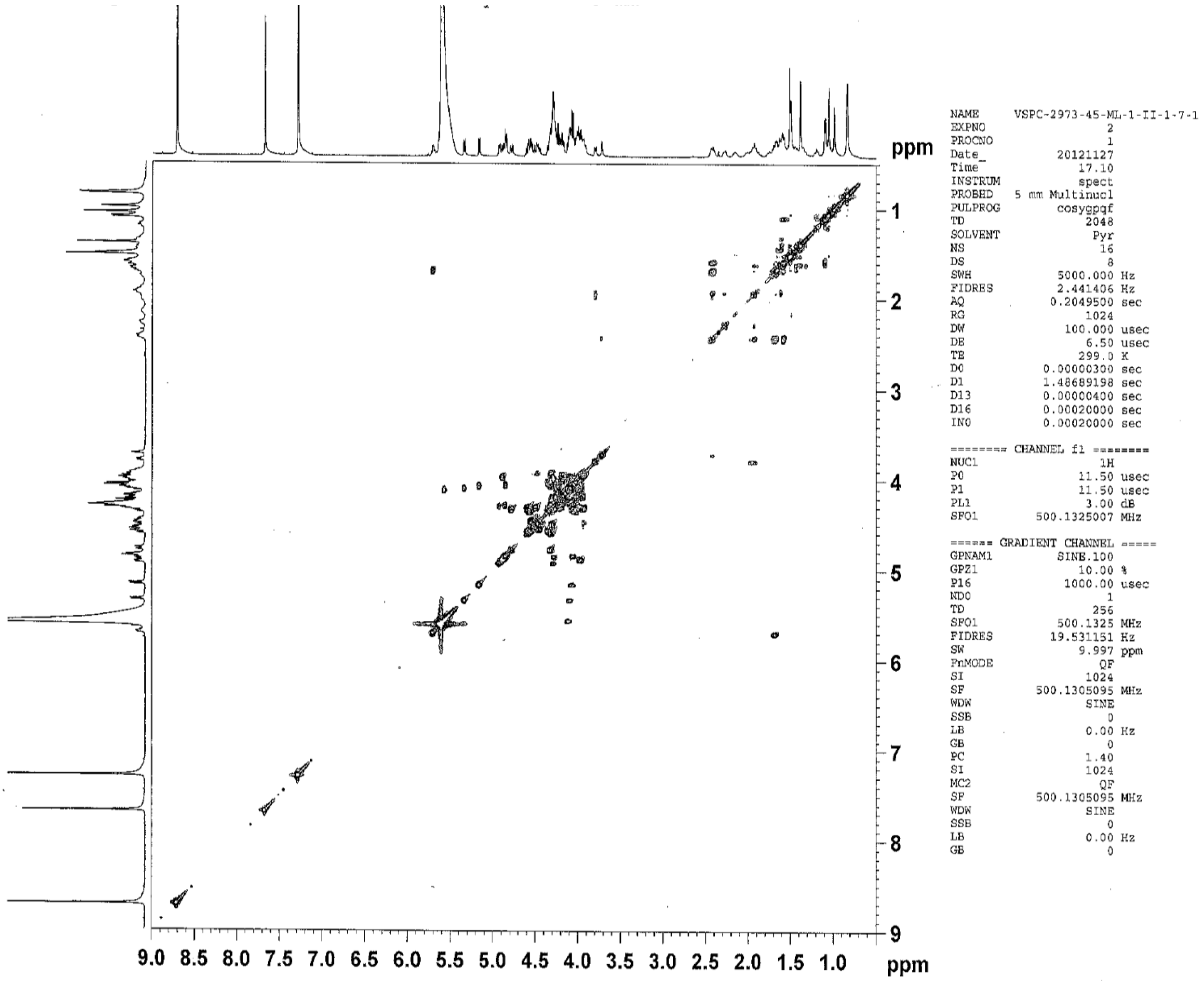

(**b**)

Figure S3. *Cont.*

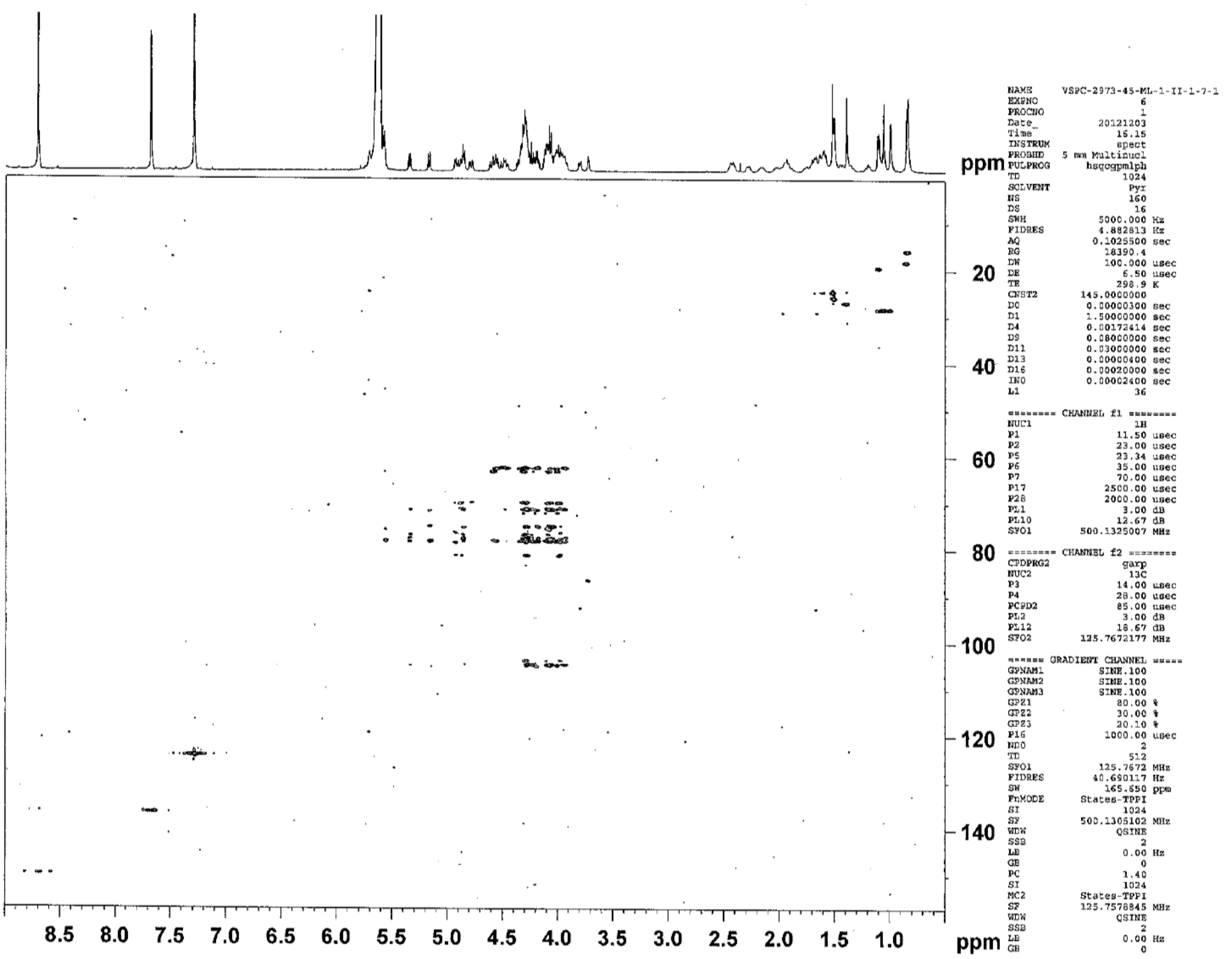

(**c**)

Figure S3. *Cont.*

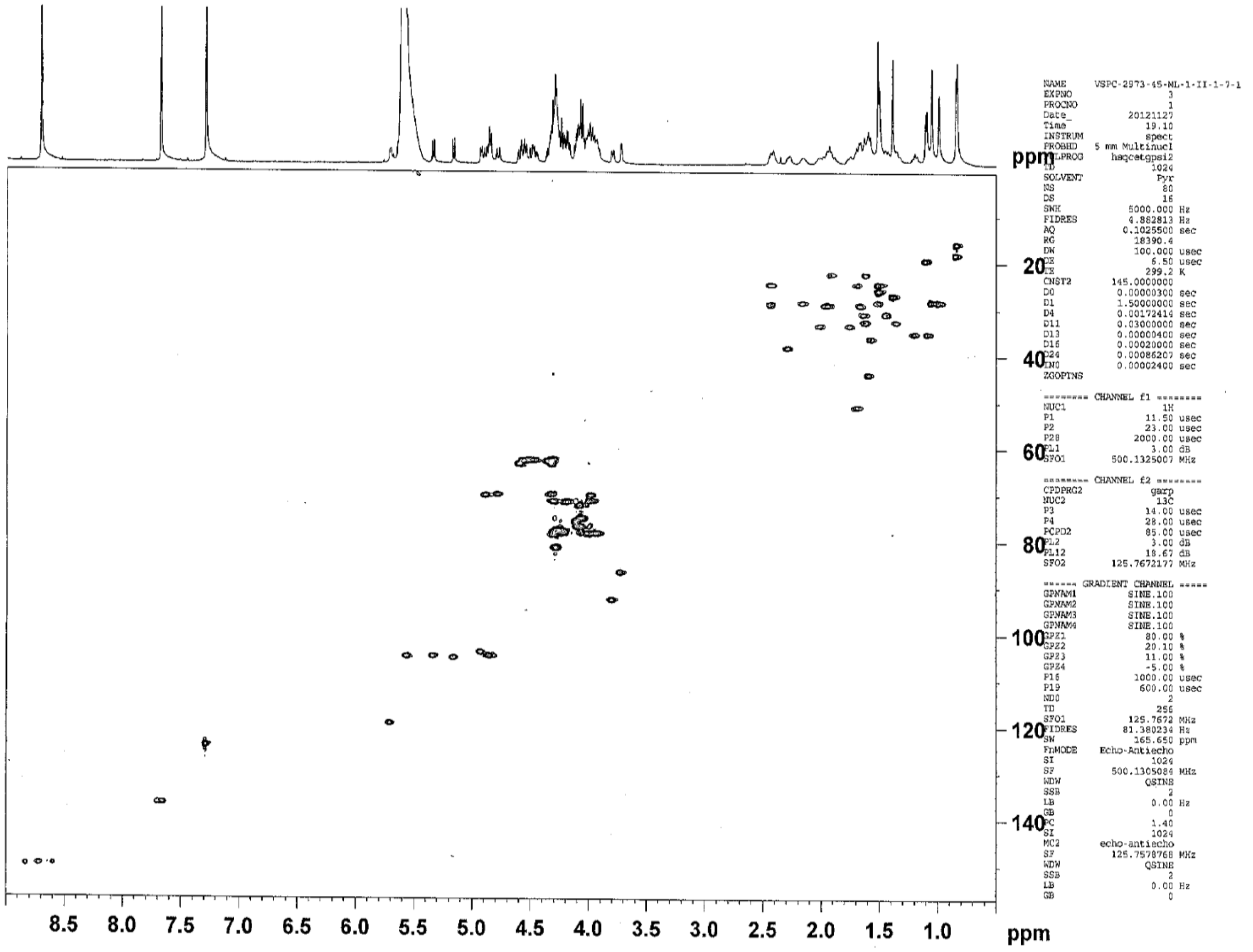

(**d**)

Figure S3. *Cont.*

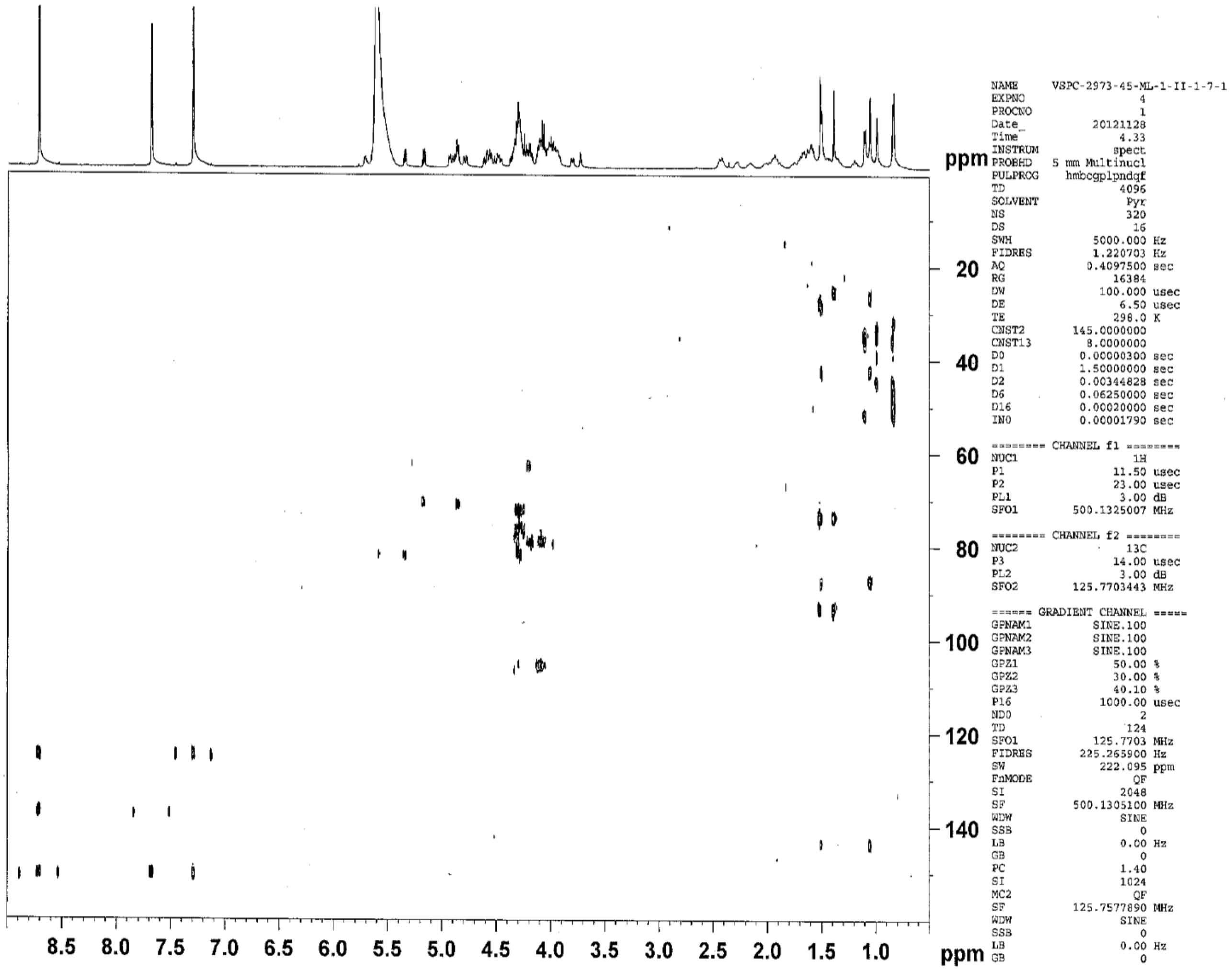

(**e**)
